# Classification of directionally specific vagus nerve activity using an upper airway obstruction model in anesthetized rodents

**DOI:** 10.1038/s41598-021-89624-3

**Published:** 2021-05-21

**Authors:** P. Sabetian, Y. Sadat-Nejad, Paul B. Yoo

**Affiliations:** 1 Institute of Biomedical Engineering, University of Toronto, 164 College St Room 407, Toronto, ON M5S 3G9 Canada; 2 Department of Electrical and Computer Engineering, University of Toronto, 10 King’s College Rd, Toronto, ON M5S 3G9 Canada; 3 Toronto Rehabilitation Institute-University Health Network, University of Toronto, 550 University Ave., Toronto, ON M5G 2A2 Canada

**Keywords:** Biomedical engineering, Preclinical research, Machine learning

## Abstract

Electrical signals from the peripheral nervous system have the potential to provide the necessary motor, sensory or autonomic information for implementing closed-loop control of neuroprosthetic or neuromodulatory systems. However, developing methods to recover information encoded in these signals is a significant challenge. Our goal was to test the feasibility of measuring physiologically generated nerve action potentials that can be classified as sensory or motor signals. A tetrapolar recording nerve cuff electrode was used to measure vagal nerve (VN) activity in a rodent model of upper airway obstruction. The effect of upper airway occlusions on VN activity related to respiration (RnP) was calculated and compared for 4 different cases: (1) intact VN, (2) VN transection only proximal to recording electrode, (3) VN transection only distal to the recording electrode, and (4) transection of VN proximal and distal to electrode. We employed a Support Vector Machine (SVM) model with Gaussian Kernel to learn a model capable of classifying efferent and afferent waveforms obtained from the tetrapolar electrode. In *vivo* results showed that the RnP values decreased significantly during obstruction by 91.7% ± 3.1%, and 78.2% ± 3.4% for cases of intact VN or proximal transection, respectively. In contrast, there were no significant changes for cases of VN transection at the distal end or both ends of the electrode. The SVM model yielded an 85.8% accuracy in distinguishing motor and sensory signals. The feasibility of measuring low-noise directionally-sensitive neural activity using a tetrapolar nerve cuff electrode along with the use of an SVM classifier was shown. Future experimental work in chronic implant studies is needed to support clinical translatability.

## Introduction

Among myriad peripheral nerve electrodes, such as spiral nerve cuffs, flat interface nerve electrodes (FINE) or intrafascicular electrodes^1–5^, have been used successfully in many nerve stimulation systems as potential means of optimizing therapies via a closed-loop controlled approach. Cuff electrodes have been shown to be stable for chronic implantation in humans for both stimulation and recording applications. However, the widespread clinical use of nerve electrodes as a means of recording peripheral nerve activity has been limited^6^. This is due to numerous factors such as (1) developing an appropriate nerve cuff to record high signal-to-noise ratio (SNR) as well as directionally-sensitive recording^7,8^ (2) clinical translation of advanced neural interfaces for long-term use in patients^9^, and (3) developing closed-loop controllers that can process and provide meaningful information about the recorded neural signals^10^.

The ability to obtain directional information from electrically measured neural activity could have important functional implications for neuroprosthetic applications, such as vagus nerve stimulation (VNS) therapy. The cervical vagus nerve is commonly referred to as the tenth cranial nerve and innervates the heart, lungs, digestive tract, and other organs of the chest and abdomen. VNS is an established therapeutic option for patients that suffer from drug-resistant epilepsy and depression, with potential for treating rheumatoid arthritis and other disorders^11^. Despite the continued use of VNS therapies in patients, the overall clinical efficacy remains modest due in part by limitations in utilizing electrically recorded vagal nerve activity as part of a closed-loop controlled therapeutic system. Importantly, it has been shown that neural signals obtained from the vagus nerve can provide a means of predicting epileptic seizures^12,13^ , as well as monitoring respiratory function^14,15^.

To date several potential techniques and methods have been developed to extract directional information from peripheral nerves. However, these techniques have been demonstrated using either arrays of multi-contacts electrode cuff (MEC) placed within a single nerve cuff electrode or using electrically-evoked neural activity (for example compound action potential (CAPs) in frog and pig^16,17^). MECs can be used to differentiate directional information by measuring the propagation of neural signals. Velocity-selective recording (VSR) methods in MECs^16–18^, have taken advantage of action potential propagation via delay-and-add operators that emphasize CAP with specific velocities and make it possible to detect the fiber type. However, this approach is unable to distinguish signals that have similar conduction velocities. Alternative method of selective recording of naturally-evoked (or mechanical stimuli) CAPs can be achieved using spatiotemporal signatures extracted from MEC recordings^19,20^. These recordings demonstrated two important concepts; firstly, that it is possible to record naturally occurring spikes using MEC and basic signal processing and, secondly, that by using either a modified VSR process or spatiotemporal signatures, it is possible to record physiological neural spikes and classify the signals based on their velocities or direction in real time. However, such MECs may not be suitable for use in small animal experiments and may likely entail complex hardware specifications (e.g., huge number of lead wires or high data transfer rate) when clinically translating this technology in patients.

Building on these ideas, our previous work showed that directionally-sensitivity recordings of KCl-evoked signals that are closer to physiological neural activity (e.g., 1–10 µV) can be achieved by using a simple tetrapolar nerve cuff electrode^21^. Tetrapolar cuff uses the absolute minimum number of electrodes needed to generate two distinct tripolar signals that, in turn, generates a low-noise, directionally-sensitive neural signal. However, the tetrapolar recording configuration is uniquely different and this study is the first application of minimizing the number of recording electrode contacts (e.g., tetrapolar nerve cuff electrode^21^).

In this paper, we present results from a study where the primary objective was to test the feasibility of using a tetrapolar nerve cuff electrode to record physiological electroneurogram signals from either the left or right vagus nerve in anesthetized rats. We employed an upper airway obstruction model to generate directional changes in neural activities (e.g., suppressed afferent respiratory fibers during obstruction)^22^. The recorded signals were classified into efferent and afferent waveforms by using a supervised learning model—support vector machine (SVM)—which used a Gaussian kernel to transform the data into latent space and differentiate neural signals based on an imposed hyperplane^23^.

## Results

### Vagus nerve activity and related physiological parameters

**Figure 1:**
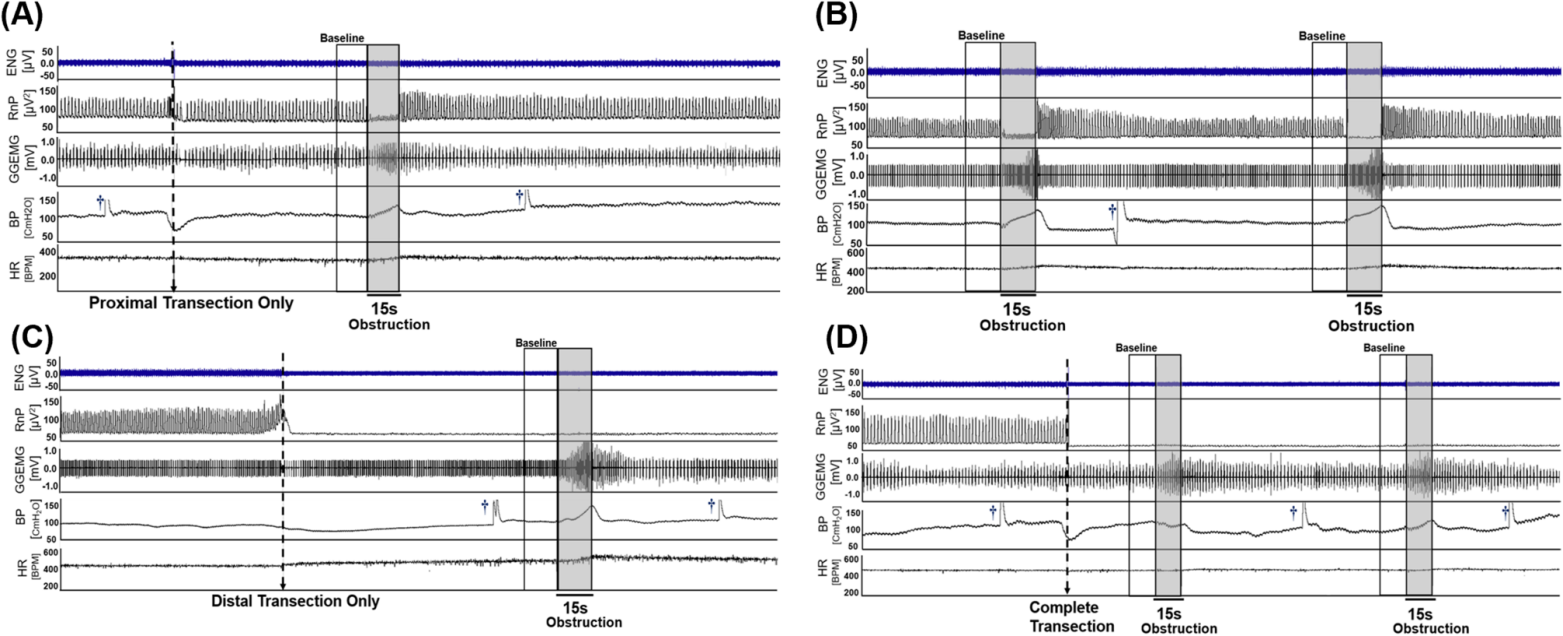
Sample data consisting of neural and physiological raw data that were measured when (**A**) left vagus nerve was intact, (**B**) left vagus nerve transected only at the proximal end, (**C**) left vagus nerve was transected only at the distal end, and (**D**) left vagus nerve was transected at both ends. It is noted that (**A**) and (**C**) are from the one experiment and (**B**) and (**D**) are from another experiment. Each signal trace (from top to bottom) represents the recorded neural activity (ENG: tetrapolar signal, RnP: respiratory-related vagus nerve profile) and physiological parameters (GGEMG: genioglossus muscle activity, BP: blood pressure, and HR: heart rate). Grey boxes represent upper airway obstructions. [†, denotes flushing of carotid catheter with heparinized saline].

**Figure 2:**
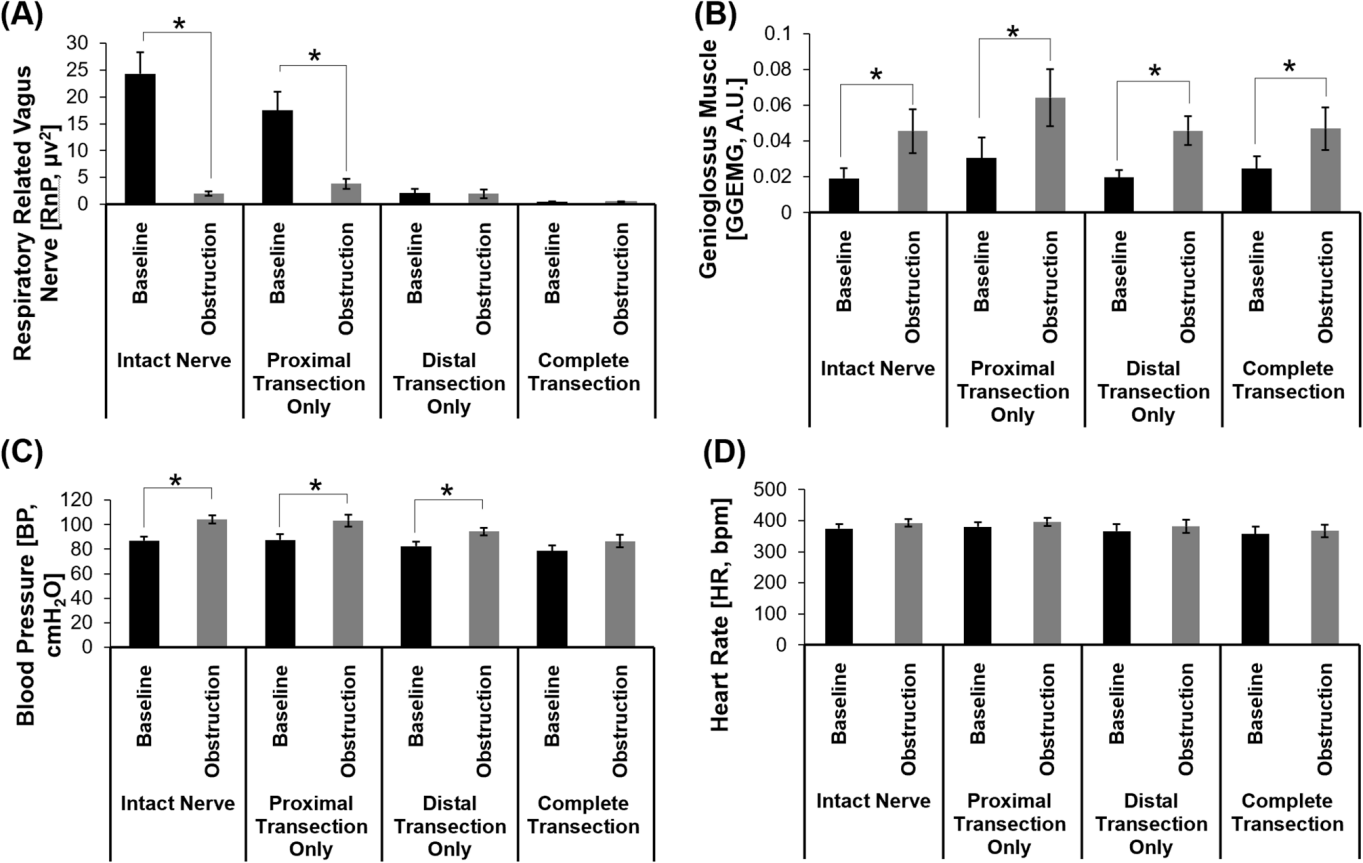
Summary of the effects of airway obstruction on different cases including (1) the intact nerve, (2) the transected nerve at the proximal end, (3) the transected nerve at the distal end, and (4) the transacted nerve at both ends. Analysis was performed for on all physiological parameters including (**A**) respiratory-related vagus nerve profiles (RnP), (**B**) genioglossus muscle activity (GGEMG), (**C**) blood pressure (BP), and (**D**) heart rate (HR). [*, *p* value < 0.05 compared to the baseline, n = 6].

In animals with an intact vagus nerve (Fig. 1A), obstruction of the upper airway resulted in an immediate and sustained decrease in the tetrapolar ENG activity (first panel, ENG [µV]) and a corresponding 92% reduction in the RnP (second panel, RnP [µV^2^]), when compared to baseline. This decrease in neural activity was accompanied by increases in GGEMG activity (138%), BP (20%), and HR (5%). The changes in variables (except for the HR) were statistically significant (Fig. 2). Once the airway obstruction was removed, all signals returned to baseline levels within approximately 1 min. Similar responses were observed in animals where the vagus nerve was only transected proximal to the recording electrode (Fig. 1B). Airway obstruction caused a significant decrease in RnP (78%) along with significant increases in GGEMG (111.3%) and BP (18%). Again, the change in HR was negligible.

Surgical transection of only the distal segment of an intact nerve (Fig. 1C) or transection of both ends of the VN (Fig. 1D) resulted in marked losses of RnP that confirmed our hypothesis that the measured neural activity primarily consisted of afferent signals traveling towards the brainstem. Naturally, there were no significant changes in RnP observed during airway obstructions (Fig. 2A), but there was a significant difference in the baseline RnP activity (p = 0.013) between distal only and complete transection of the VN (Fig. 1C vs. 1D, respectively). Compared to the absence of RnP activity (Fig. 1D), there was a measurable amount of efferent VN activity when the proximal end remained intact, however airway obstructions did not cause any changes in efferent neural output.

With only the distal VN transected (Fig. 1C), airway obstructions elicited significant increases in GGEMG (133.3%) and BP (15%), with negligible changes in HR. Both responses returned to baseline shortly (e.g., ~ 1 min) after re-opening the upper airway. Interestingly, with the VN completely transected (Fig. 1D), there was only a significant increase in GGEMG (91%).

### Accuracy of classification models

**Figure 3:**
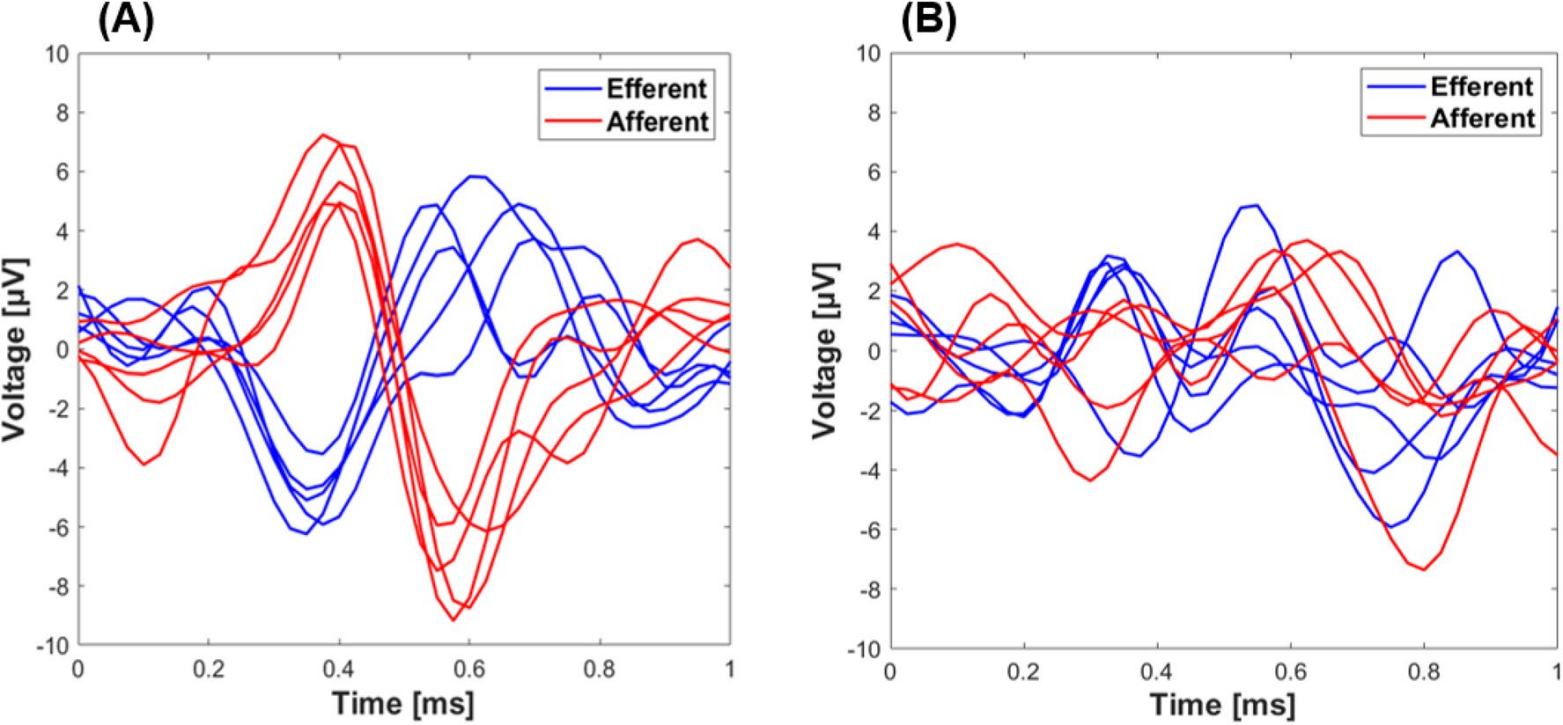
Sample data of neural recordings obtained from a single experiment, where the vagus nerve was transected only proximally (afferent) or distally (efferent) from the nerve cuff electrode. Signals representing (**A**) synchronized and (**B**) asynchronized ENG signals were plotted in 1 ms windows. [x-axis: time = 0–1 ms; y-axis: amplitude =  − 10–10 μV].

Samples of 6 raw data of afferent (proximal transection only) and efferent (distal transection only) activity from either the left or right VN of one experiment is shown in Fig. 3. One can see that the main difference between the two waveforms is the opposite polarity, where afferent and efferent signals were completely synchronized (Fig. 3A). However, due to the amount of noise within the data and the complexity of the waveforms (e.g., the starting point of the AP and the possibility of overlap, refer to Fig. 3B), more advanced methods such as machine learning approaches are required to classify afferent from efferent waveforms. As mentioned in Methods and Materials, a thresholding method was used to denoise the data and find the peaks that correspond to the ENG waveforms. The average of the threshold value among all experiments was obtained 2.8 ± 0.3 µV.

**Figure 4:**
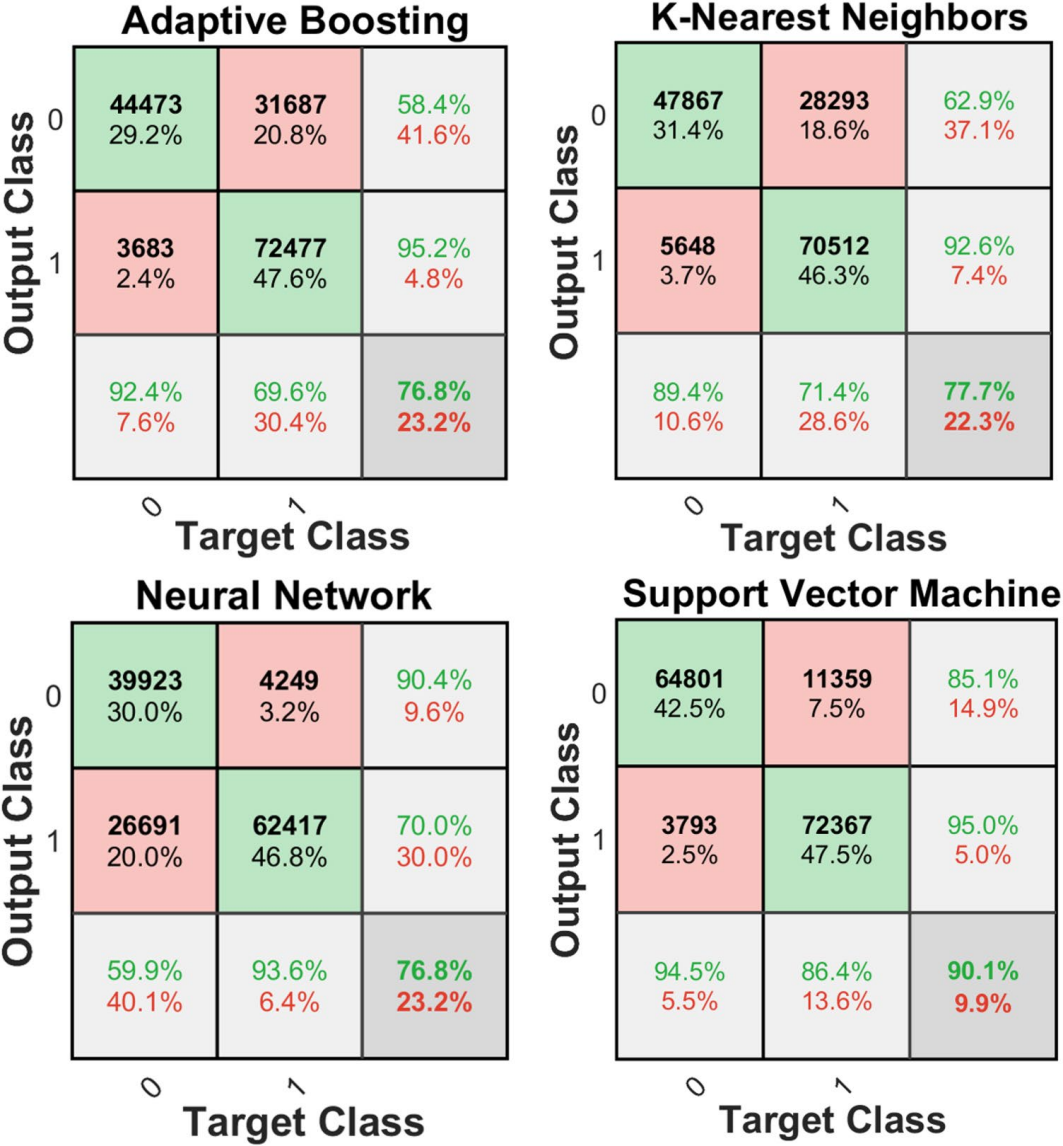
Confusion matrices corresponding to the different algorithms used for the 2-class problem constructed with combined test sets (n = 6 rats). This data was tested on Model 1 only. The last row and column of each confusion matrix show the recall and precision per class (class0: efferent, class1: afferent), respectively.

**Figure 5:**
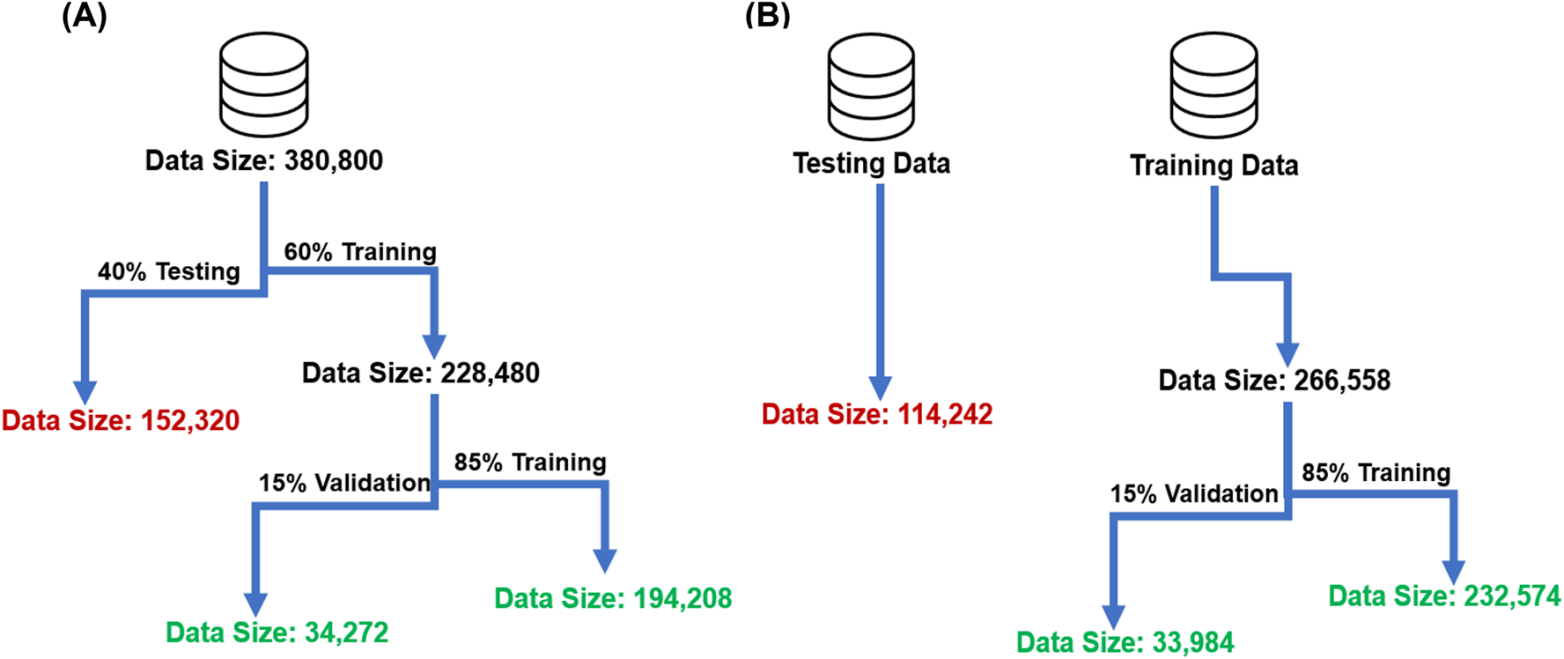
Illustrates the flow chart of (**A**) Model 1 and (**B**) Model 2. (**A**) In model 1, all data from every experiment were concatenated and then randomly separated into two different data sets of (1) training and (2) testing by considering a ratio of 60% to 40%, respectively. The training set was further split into 85% training and 15% validation sets. (**B**) In model 2, one experiment was randomly chosen for testing, while the rest of the data was used for training and validation with a ratio of 85% to 15%, respectively.

The confusion matrix of the mentioned classifiers (refer to Machine Learning in the Methods and Materials) was calculated and compared for all of the 4 different classifiers for 2-class classification [e.g., class0 (efferent) and class1 (afferent) in model 1 (Fig. 4)]. Furthermore, the summary of F_1_-score for all the classifiers using model 1 applying validation set (refer to Fig. 5) is shown in Table 1. Both the confusion matrix as well as the F_1_-score analysis show that the SVM classifier exhibits the best performance among all the classifiers for the 2-class classification of this study’s raw data. Therefore, the SVM classifier was chosen as the classifier for both models (e.g., model 1 and model 2).

**Table 1:** Summarizes the F_1_- score for different algorithms applied to the 2-class problem (efferent and afferent) using Model 1.

F_1_-score	SVM	Neural network	K-nearest neighbours	Adaptive boosting
Efferent	0.8472	0.7403	0.7383	0.7155
Afferent	0.8462	0.6721	0.8060	0.8039

Confusion matrix analysis for 2-class classification showed a significant imbalance in accuracy in two different classes (e.g., 94.5% for the class 0 vs. 86.4% for the class1 in the SVM classifier, refer to Fig. 4). To tune hyperparameters (cost function penalty term in this case), the validation set was used as described in Methods and Materials section. This method demonstrates a ratio of 1.5:1 for afferent and efferent respectively, which leads to a more balanced prediction between the classes for the selected SVM model. This means that misclassifying afferent data leads to a higher penalty for the classifier. Based on this new ratio, the SVM classifier was reconstructed with the balanced classification model (e.g., the ratio of 1.5:1 for afferent and efferent, respectively).

**Figure 6:**
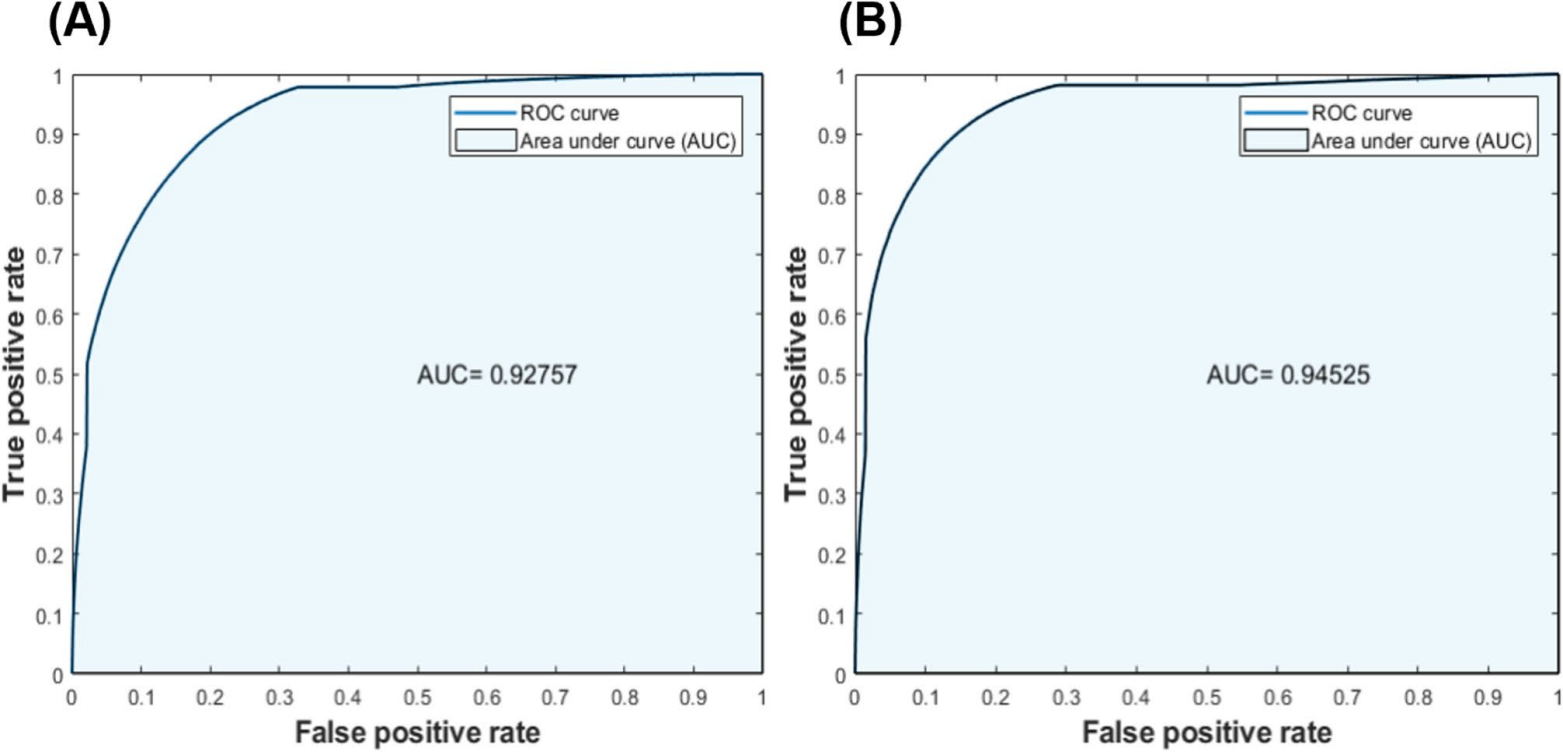
Receiver operating characteristic (ROC) and the area under the curve (AUC) for the calculated the performance of both classification models. These plots were conducted with respect to the true positive rate and false positive rate of the test set; (**A**) shows the ROC for the classification model1 and (**B**) shows the ROC for the classification model 2. The obtained results in both parts illustrate that both models had an acceptable performance in distinguishing the two different classes, with an AUC of 93% and 95%, respectively.

The performance of both proposed models (e.g., model 1 and model 2) in differentiating two classes [e.g., class 0 (efferent) vs. class 1 (afferent)] was further evaluated using ROC and AUC techniques (Fig. 6). The ROC plot illustrates a value between zero and one, where an AUC of one refers to a perfect separation of the classifier between the two classes, and an AUC of 0.5 represents chance. True positive rate (or the y-axis) illustrates the rate at which samples were correctly classified, while false positive rate (or the x-axis) illustrates the portion of the samples that were misclassified. Both Figs. 6 A and B show an overall AUC of 93% and 95%, respectively. These results confirm two important points: (1) both models have high accuracy in the detection between the two classes and (2) both models perform similarly. The only difference between the two models is the ratio of training and validation data sets.

**Figure 7:**
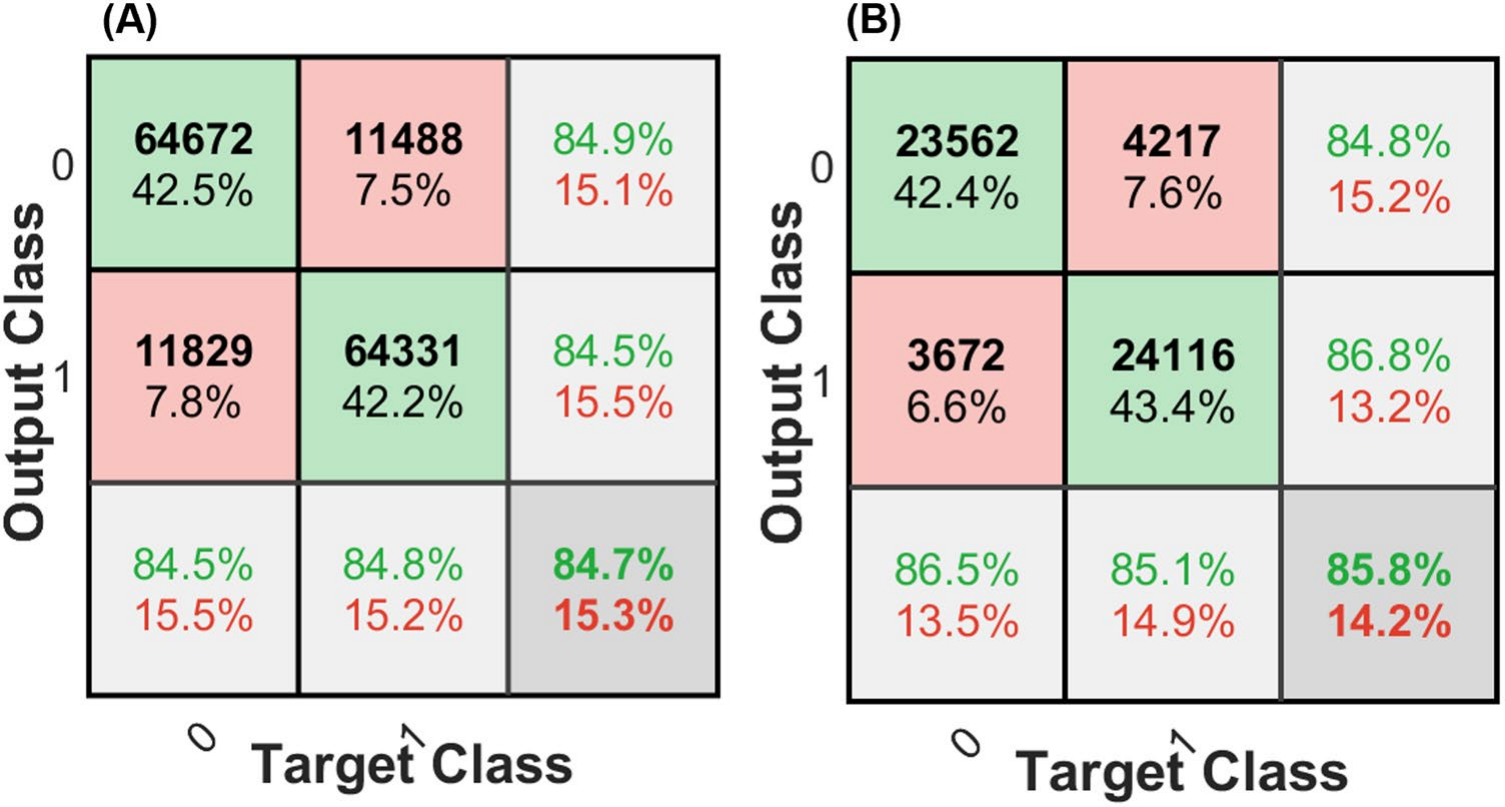
Confusion matrices corresponding to the testing data used to distinguish class 0 (efferent) from class1 (afferent) ENG data: (A) Model 1 and (B) Model 2. Both figures illustrate a high performance of both models in detecting efferent or afferent data and there is a close balance on performance that confirms the generalization and robustness of both models. The last row and column of each confusion matrix shows the recall and precision per class, respectively.

The confusion matrices obtained for both model 1 and model 2 illustrate the performance of the SVM classifier on each individual class, as shown in Figs. 7 A and B, respectively. Model 1 achieved accuracies of 84.5% and 84.8% for class 0 (efferent) and class1(afferent), respectively (Fig. 7A). Model 2 had accuracies of 86.5% and 85.1% for class 0 and class1, respectively. Overall, model 1 and model 2 had accuracies of 84.7% and 85.8%, respectively for 2-class classification. These high accuracies as well as the similarity between these two models suggests that the features used and the learned classifier led to a generalizable and robust classifier. The main difference between model 1 and model 2 refers to their test sets. Model 1 was tested on the unseen individual points but from experiments that the model was trained on. While, in model 2, the test set was chosen from a set of new data points from an unseen experiment. The non-significant and negligible difference (e.g., only 1.1%) in accuracy of these two models could be due to variation in SNR.

### Hypothesis testing using airway obstruction data

**Figure 8:**
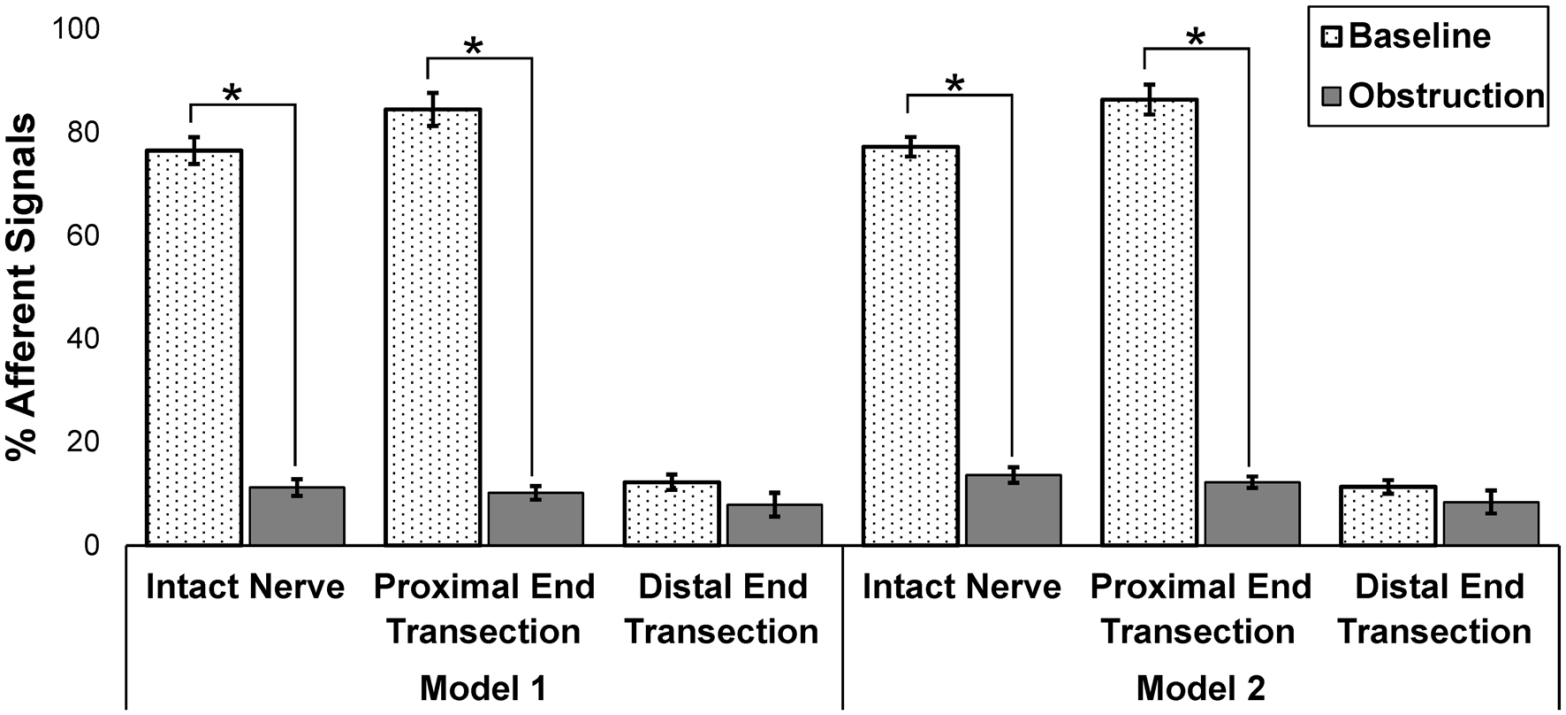
Comparison of the % afferent signals recorded during both baseline and airway obstruction for cases of (1) intact nerve, (2) transection of only the proximal end, and (3) transection of only the distal end. Analysis was performed for model 1 and model 2 classifiers. [*, *p* value < 0.05 compared to baseline, n = 6].

In order to further test the accuracy of both model 1 and model 2, the obstruction data that was collected for the intact nerve, the proximal end transection, and the distal end transection were fed to both classifiers so as to evaluate the outcomes. All data was then compared to their corresponding baseline data. Both models predicted that the percentage of afferent signals significantly decreased for both the intact nerve (60% decrease) and the proximal transection only (70% decrease), during obstruction. However, this value did not change significantly (e.g., 5% decrease) during obstruction in the case where only the distal VN was transected (Fig. 8). This data confirmed the hypothesis that during obstruction the majority of the afferent neural activities are suppressed, and the majority of neural signals consisted of efferent activity. This data also verifies the suitability in performance of our classifiers in detecting afferent and efferent signals.

Both classifiers illustrate that, when compared to baseline, afferent signals decreased significantly during obstruction for both the intact nerve and after the proximal end was transected. This value did not change significantly in the case where the vagus nerve was transected distal to the recording electrode.

## Discussion

In this study, we present preclinical data that supports the feasibility of using a single nerve cuff electrode in conjunction with a machine learning algorithm to differentiate VN signals travelling in opposite directions. Using an anesthetized rodent model of obstructive sleep apnea, changes in neural activity (RnP) and relevant physiological signals (HR, BP, and GGEMG) were assessed under four different experimental conditions: (1) an intact nerve, (2) nerve transected at the proximal end of the cuff electrode, (3) nerve transected at the distal end, and (4) nerve transected at both ends. Our experimental results showed that a tetrapolar nerve cuff electrode can be used to measure VN activity in a directionally-specific manner. Afferent and/or efferent neural activity was compared between baseline conditions and during brief episodes of airway obstructions, where significant decreases in afferent RnP were observed under certain conditions (e.g., proximal nerve transection). As predicted by previous work in other animal models^18,22,24,25^, we observed significant increases in GGEMG activity (range = 91–138%) and BP (range = 15–20%) that were associated with airway obstructions. It is noted that the GG muscle responded to obstructions, regardless of whether the VN was intact or bilaterally transected. In contrast, there were no changes in BP in response to airway obstructions when the VN was transected bilaterally. Considering that there were no significant changes in HR (e.g., tachycardia), it is likely that the observed increases in BP were mediated by vagal afferents.

The results of these experiments suggested an interesting model to test the bi-directional ability of a tetrapolar nerve cuff electrode in recording VN activity. After filtering, thresholding and labeling all raw ENG (e.g., VN APs) the data was fed to a machine learning algorithm which was trained with 380,800 examples of signals corresponding to afferent and efferent labels. To ensure that ECG and EMG artifacts were not mistaken for the CAPs^19^, a sample size window of 1.025 ms (less than 5 ms) was set for the individual CAPs. In this study, support vector machine (SVM) with Gaussian kernel^23^ was chosen as the classifier since it had the best performance among other classifiers such KNN with coarse kernel^26^, Adaboost^27^, NN with one hidden layer and 10 units^28^, and were used to perform classification learning (refer to Fig. 4).

Two different models were trained in SVM with variation in training, validation and testing ratio. In the first model, all data from every experiment were concatenated and then randomly divided into training (60%) and testing (40%) sets. In which, training data was further divided into 85% training and 15% validation. In the second model, one of the experiments was set aside, and the remaining data were trained to a validation ratio of 85% to 15%, respectively for tuning hyper parameters. Models 1 and 2 illustrated accuracies of 84.7% and 85.8% on the testing set. While both models achieved high accuracy on the testing set, model 2 yielded about 1% higher accuracy (84.7% vs. 85.8%) compared to the model 1. This was achieved with a smaller number of training data compared to model 1 (e.g., 228,480 vs 161,814). The difference in accuracy could be attributed the testing data in model 2 being obtained from an experiment with high SNR, whereas the testing set in model 1 were randomly selected from experiments with variable SNR values. In conclusion, having high accuracy in both models illustrates that the model (e.g., used features and classifier) are robust and generalizable. However, it needs to be mentioned that the prediction of models is sensitive to the quality of recording. This means that if the testing data experiment had low SNR, the prediction would be lower, as the model may have been trained on higher-quality experiments. In this study, however, we were able to show that reliable differentiation between efferent and afferent signals could be achieved using neural activity with relatively low SNR (6.05 ± 0.36 dB, range: 4.3–7.3 dB). It is reasonable to predict that the performance of machine learning algorithms could be further improved with higher SNR signals (e.g., CAP) recorded from peripheral nerves.

In the final part of this study, the performance of both models was tested using a new data set from obstruction recordings that were fed to both model classifiers. Both models predicted obstruction data as efferent signals with above 80% confidence, which confirms that the obstruction includes efferent data. As noted above, the obstruction data should theoretically consist of efferent nerve activity. However, less than ideal SNR of the recording data as well as the existence of overlapping afferent and efferent data could have caused the obstruction data model to not consist of purely efferent activity. In addition, the upper airway was mechanically occluded for only short periods of time and that an incomplete occlusion in some trials could have affected the results in terms of yielding purely efferent or afferent activity during obstruction.

Thus far, several techniques and algorithms have been developed to improve the recording selectivity of extra-neural electrodes. These techniques revolve around two general approaches: (1) using the temporal information of recorded neural signals such as velocity-selective recording (VSR) methods^16–18,29^ or (2) using the spatial and/or spatiotemporal characteristics of neural signals to localize the sources within a compound nerve trunk^19,20,30,31^. Most of these studies involved CAPs that were electrically evoked using stimulation electrodes^16,32–34^, leading to much higher amplitude signals than naturally evoked neural activity (e.g. produced by proprioceptive or mechano-sensory afferent activity). For studies involving naturally evoked afferent activity^18,35^ classification was applied to windowed signals (e.g. rectify-bin-integrated), not to individual CAPs. While a major advantage of multi-contact electrode arrays is that they can be used to easily tune the recording system to differentiate the fiber-type among the multitude of recorded neural signals, the need for large numbers of recording sites may require relatively longer nerve cuff electrodes. Large multi-electrode arrays may not be suitable for use in small animal experiments and may likely entail complex hardware specifications (e.g., large number of lead wires or high data transfer rates) when clinically translating this technology in patients.

The results of this study suggest a minimum number of recording sites needed in a nerve cuff electrode that when combined with machine learning techniques (e.g., SVM classifier) can effectively distinguish efferent and afferent information from physiologically evoked neural activity. The results suggest that good performance of directional recording may be achievable with fewer number of contacts and may be used as a template for designing more complex implantable nerve cuff electrodes with greater geometrical efficiency.

Further refinements to classification algorithms such as applying cross validation in model 2 to ensure the model will provide consistent results as that using different experiment for testing data set, combined with improvements to the SNR and AP detection, could likely yield more robust discrimination performance and improved tracking of neuronal firing rates. Future in vivo studies should investigate the long-term performance of tetrapolar nerve cuff electrodes. The potential improvements in monitoring physiological function could lead to precise control signals for assistive devices that are able to produce more natural movements and more efficient electrical neuromodulation systems.

Implementing electrical neuromodulation therapies in a closed-loop manner could have a significant impact on the use of implantable neurostimulators for treating patient populations. A logical progression of the current work is to explore the use of tetrapolar recording configurations in high density nerve cuff electrode arrays designed to be implanted around peripheral nerves. Recent preclinical studies suggest that VNS could potentially be used to treat drug-resistant hypertension by modulating baroreflex depressor responses^36,37^. It has also been shown experimentally that afferent vagal nerve activity can serve as a physiological biomarker of BP^38^, which in turn could provide a feedback mechanism for adjusting VNS parameters. As shown by our classification results, a tetrapolar nerve cuff can be used to record low-noise afferent or efferent neural activity to track changes in a variety of physiological functions, such as those of the respiratory, cardiac or vascular systems.

## Methods

### Surgical setup

Acute, non-survival in vivo experiments were conducted in **6** Sprague Dawley rats (male, weight = 500–750 g). All surgeries and procedures were approved by the University of Toronto Animal Care Committee in accordance with the regulations of the Ontario Animal Research Act (Toronto, ON, Canada). The design and conduct of the study were in accordance with the ARRIVE guidelines. Anesthesia was initially induced by inhaled isoflurane (5% in 100% O_2_) within an induction chamber and subsequently maintained with a gas mask (2–3% isoflurane, O_2_ flow rate:1 L/min). Following completion of all surgical procedures, the anesthesia was transitioned to urethane (initial SQ bolus of 1.2 g/Kg body weight followed by supplemental doses of 0.1–0.2 g/Kg in 1 ml of sterile saline). Body temperature (37–39 °C), heart rate (300–400 beats min^−1^), and blood O_2_ level (98–100%) were monitored and maintained throughout the experiments.

**Figure 9:**
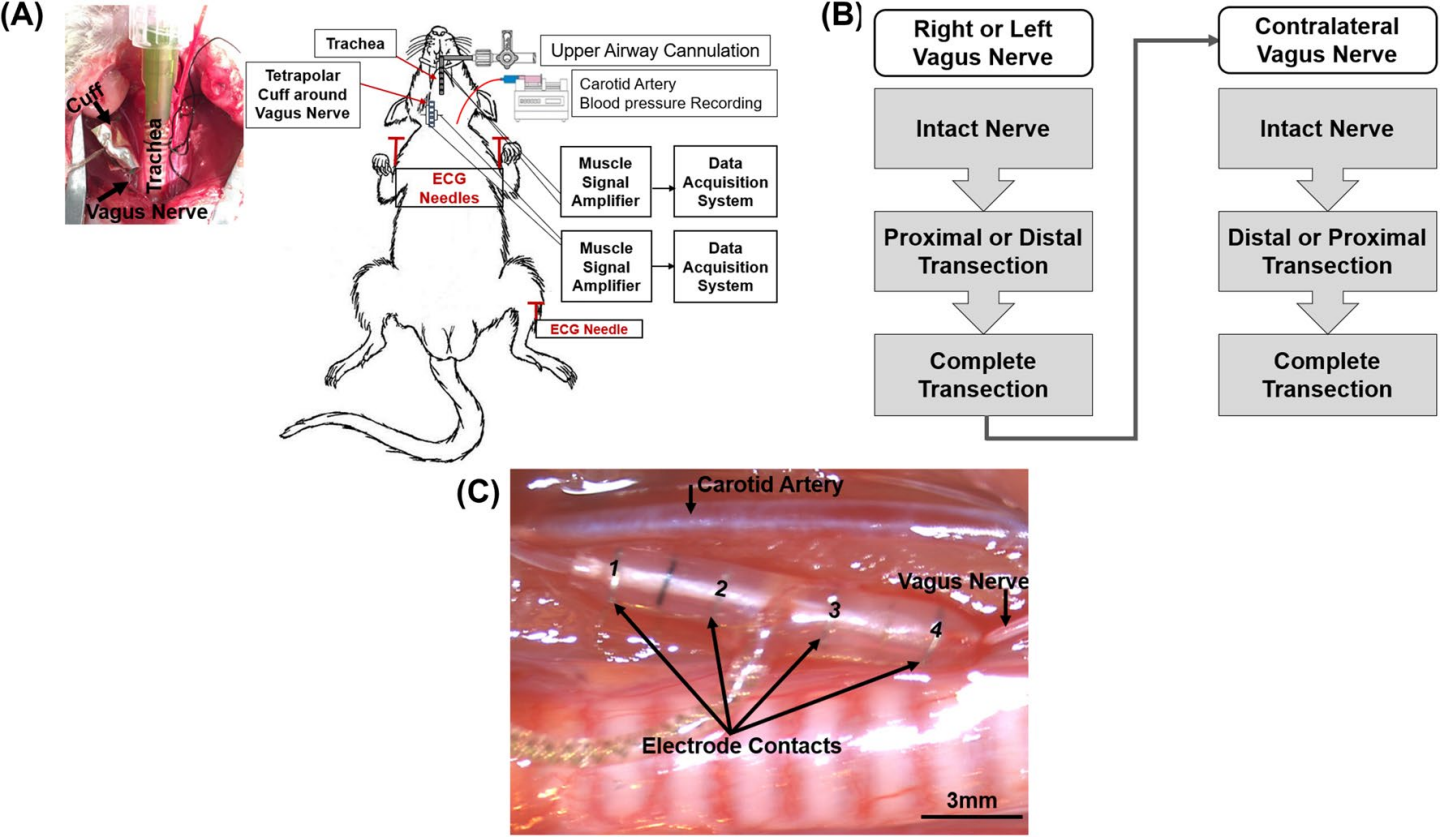
Overview of experimental setup. (**A**) Image of rat upper airway instrumented with tracheal tube along with a schematic of apparatus used to obtain physiological signals**.** (**B**) Flow chart that illustrates the recording protocol from either the left or right vagus nerve, and (**C**) A tetrapolar nerve cuff electrode implanted on the left vagus nerve (diameter = 500 µm, inter-electrode distance = 2.5 mm, and total cuff length = 12 mm).

With the rat in the supine position, a midline ventral incision and blunt dissection of neck muscles provided access to the trachea immediately below the larynx. Following a lateral incision made between two adjacent rings of tracheal cartilage, a tube was inserted in the caudal direction (blunt needle, 14G). The proximal end of the tube was connected to a 3-way stopcock, which was used to create upper airway occlusions. The vagus nerve was dissected and a tetrapolar nerve cuff electrode (NC-0.5-4-100P-2.5, inner diameter = 500 µm, 4 contacts, 100 µm wide platinum contact, inter-electrode distance = 2.5 mm, and cuff length = 12 mm, Microprobes Inc. Gaithersburg, MD, USA) was implanted (Fig. 9). The electroneurogram (ENG) was measured as 2 independent bipolar recordings (contacts 1 & 4, contacts 2 & 3) that were subsequently converted to a single tetrapolar ENG signal. The outer surface of the recording electrode was wrapped with a layer of aluminum foil (thickness = 0.025 mm) for the purpose of reducing noise artifacts^39^. The carotid artery was catheterized with PE-50 tubing (Intramedic Polyethylene Tubing, BD Medical, Canada) to measure blood pressure. The catheter was connected in series with a pressure transducer (Deltran, Model: DPT-100, Utah Med, Midvale, USA) and a syringe filled with heparinized saline (0.5 IU/ml). The syringe was controlled with an infusion pump (Pump11 Elite Infusion, Harvard Apparatus, Holliston, USA). The electrocardiogram (ECG) was obtained by inserting needle electrodes (23G, length = 1″) in the forepaws and left hind paw. A pair of stainless-steel wires (diameter: 0.003″ Bare, 0.0055″ coated; length: 100 Feet; A-M systems, USA) were desheathed and inserted into the genioglossus muscle to record the electromyographic activity of the tongue muscle (GGEMG). All electrical signals were amplified (gain = 1000), filtered (ENG = 100 Hz to 10 kHz, and EMG = 30 Hz–3 kHz, SR560, Stanford Research Systems, Sunnyvale CA), and digitally recorded (sampling rate = 40 kHz, Powerlab 16/35, ADInstruments, Colorado Springs, CO). A needle inserted in the lateral abdominal fat pad served as the electrical ground.

### Experimental protocol

Multiple physiological parameters (BP, ECG, and GGEMG) as well as VN activity were measured continuously throughout each experiment. Following a 1-h acclimation period, the experimental protocol consisted of (1) a baseline period, (2) series of five obstructive apneas, each with a duration of 15 s and separated by 1 min, and (3) post obstruction period (refer to Fig. 9B). The protocol was performed with the VN intact, repeated after transecting the nerve either proximal (i.e., above) or distal to the recording electrode, and repeated again after transecting the remaining intact end of the nerve. In each animal, this protocol was performed on both the left and right vagus nerves in randomized order. The animal was euthanized at the end of each experiment by intra-cardiac injection of 0.3 ml/Kg T-61 (Merck Animal Health, Kirkland, QC, Canada).

Changes in cardiovascular function parameters (e.g., HR and BP) were quantified in 15-s time bins during the baseline, obstruction and post-obstruction periods. The HR was calculated by averaging the time interval between successive R-peaks and the BP was obtained by calculating the average pressure (Lab chart Pro v8.1.5, ADInstruments, Colorado Springs, CO). Processing of the GGEMG data involved signal rectification, filtering (triangular Bartlett filter, window width = 300 ms) and then applying user-specified adaptive threshold methods (e.g., findpeaks, threshold = 0.2 mV). The GGEMG signal was quantified by the area under each detected peak. Signal to noise ratio (SNR) of neural activities was defined by the log ratio of the signal to the baseline noise (1):

1 $$ SNR = 20{\text{log}}\left[ {\frac{{Vpp\left( {Signal} \right)}}{{Vpp\left( {Noise} \right)}}} \right] $$

### Signal processing and data analysis

#### Afferent/efferent spike extraction from ENG signals

The mathematical expressions of the three different ENG recording configurations are provided below: tetrapolar (Eq. 2), (Eqs. 3–4), and bipolar (Eqs. 5–6). We converted the basic definition of the tetrapolar ENG (Eq. 2) into an algebraic function of two bipolar ENG signals (Eq. 9) by calculating the difference between the two tripolar expressions (Eq. 7 through 9):

2 $$ Tetrapolar = Tripolar 1 - Tripolar2 $$

3 $$ Tripolar 1 = V2 - \left[ {\frac{V3 + V1}{2}} \right] $$

4 $$ Tripolar 2 = V3 - \left[ {\frac{V2 + V4}{2}} \right] $$

5 $$ Bipolar1 = V1 - V4 $$

6 $$ Bipolar2 = V3 - V2 $$

7 $$ Tetrapolar = \left[ {V2 - \frac{V3 + V1}{2}} \right] - \left[ {V3 - \frac{V2 + V4}{2}} \right] $$

8 $$ Tetrapolar = \frac{1}{2}\left[ {V1 - V4} \right] + \frac{3}{2}\left[ { V3 - V2} \right] $$

9 $$ Tetrapolar = \frac{1}{2}\left[ {Bipolar1} \right] - \frac{3}{2}\left[ {Bipolar2} \right] $$

**Figure 10:**
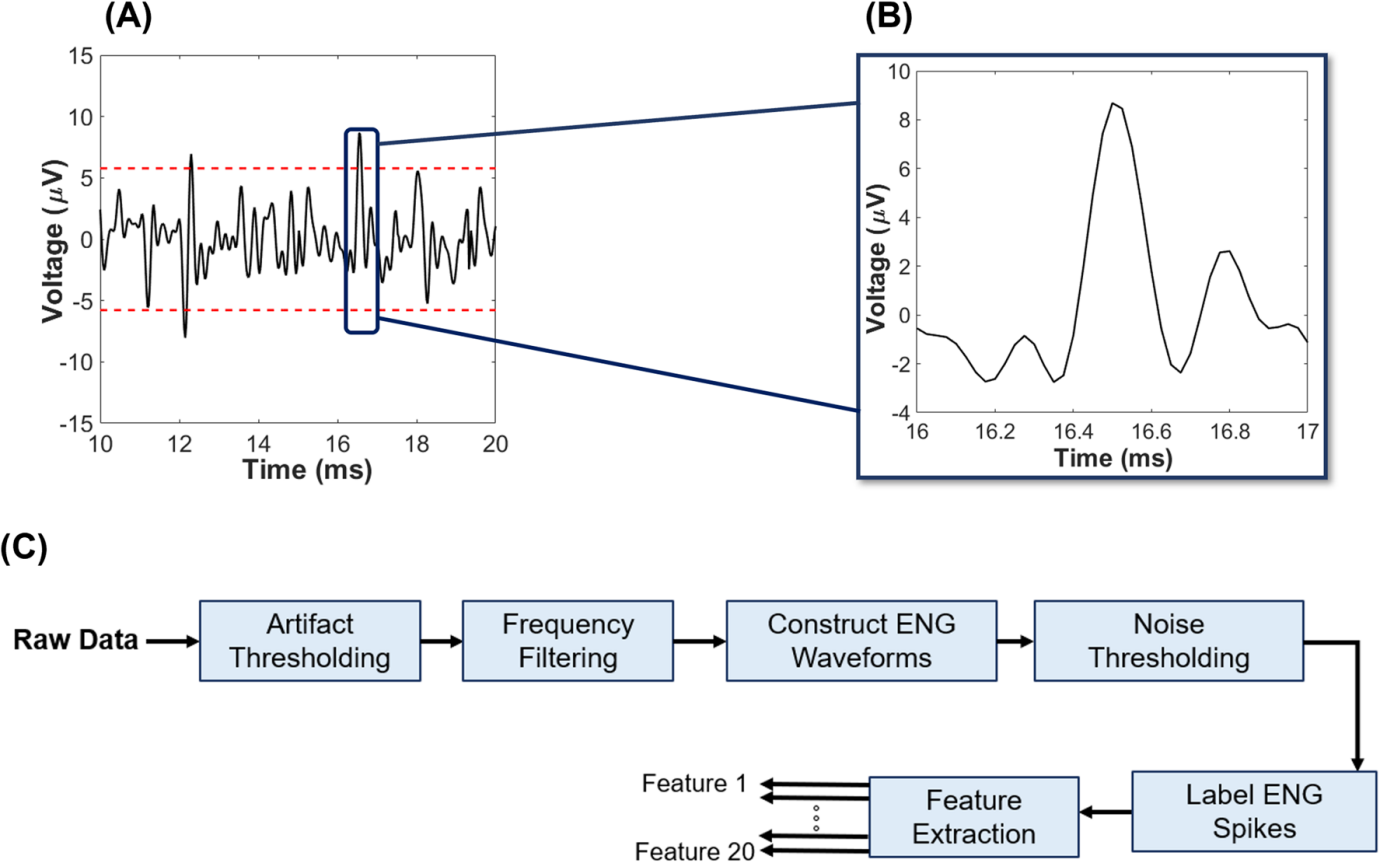
Process of extracting ENG signal. (**A**) An example of thresholding applied to raw ENG data, where the dashed lines represent ± 3δ deviation from the mean assuming purely uncorrelated additive white Gaussian noise sources (absolute value = 2.8 ± 0.3 µV). (**B**) Zoomed image of a single action potential that was recorded from the distal nerve stump (i.e., afferent) using a tetrapolar cuff electrode. (**C**) Steps applied to all raw data leading to feature extraction. The threshold in ‘Noise Thresholding’ block is constructed using a global representation of the noise, which was recorded from ENG channels with completely transected vagus nerve (e.g., absence of neural signal) and averaged across all experiments. The first three blocks (e.g., ‘Artifact Thresholding’, ‘Frequency Filtering’, and ‘Construct ENG Waveforms’) were applied to signal as well as the noise that is used for the thresholding process.

Bipolar signals were obtained from animal experiments and initially processed via hard-thresholding (threshold set at 700 µV) to remove any large-amplitude artifacts (‘Artifact Thresholding’, Fig. 10C). An 8th-order Butterworth bandpass IIR filter (passband = 250 Hz and 10 kHz,^18^) was applied to the bipolar signals (‘Frequency Filtering’, Fig. 10C) and then subsequently used to construct the tetrapolar (ENG) signals (‘Construct ENG Waveforms’, Fig. 10C). The respiratory related nerve profile (RnP) was computed by filtering (bandwidth = 250 Hz–10 kHz) the tetrapolar ENG signal, mathematically squared to obtain the signal power, and then low-pass (2 Hz) filtered^14^ as shown in panel 2 of Figs. 1A–D.

ENG signals were further processed by limiting the amplitude of the expected neural signal to be less than or equal to three-times of standard deviation of noise (‘Noise Thresholding’, Fig. 10A,B), which was assumed to consist of uncorrelated additive white Gaussian noise^40^ The actual noise used in this model was obtained from ENG measurements taken after the vagus nerve was transected both above and below the recording electrode (refer to Table 1). The formula used to calculate the noise threshold is shown as follows:

9 $$ T_{upper} = mean\left( {Data_{Tetra} } \right) + 3std\left( {Noise_{Tetra} } \right) $$

10 $$ T_{lower} = mean\left( {Data_{Tetra} } \right) - 3std\left( {Noise_{Tetra} } \right) $$

Considering the duration of neural signals and data sampling rate (e.g., 40 kHz), the peak value of each identified ENG signal was centered within a 41 sample (= 1.025 ms) time window and used to capture nerve action potentials (‘Label ENG Spikes’, Fig. 10C). Each window was labeled in two different categories (e.g., afferent or efferent) depending on its recording category (Table 2) and subsequently used in training the machine learning algorithm (Feature Extraction, Fig. 10C). All processes were performed in Matlab (Mathworks Inc., Natick, MA, USA).

**Table 2:** Summarizes the efferent or afferent ENG activity associated with different paradigms of ENG recording.

Pre-obstruction	Obstruction	Post-obstruction	Proximal transection only	Distal transection only	Complete transection
Afferent + efferent	Afferent + efferent (dominant)	Afferent + efferent	Afferent	Efferent	Noise

#### Machine learning

The machine learning framework was designed with the objective of classifying afferent and efferent ENG signals. Specific data sets were used to build a classifier that could categorize purely efferent and afferent waveforms (refer to Table 2). The procedure of windowing and labelling of the data was repeated for multiple obstructions, from which we sampled a total of 380,800 labelled spikes. A total of 190,400 afferent signals (recorded while only the proximal end of VN was transected) and 190,400 efferent signals (recorded while only the distal end of VN was transected) were used for machine learning classification. The algorithm was trained by using 20 different features related to efferent and afferent signals (Table 3).

**Table 3:** List of features that used in training algorithm.

1. Variance	2. Skewness
3. Kurtosis	4. Entropy
5. Maximum absolute value of Fourier Transform (fft)	6. Root Mean Square value (RMS)
7. Average mobility^41^	8. Range defined as maximum subtracted minimum value
9. Average power	10. Average of first derivative
11. Number of zero-crossing	12. Sign of maximum peak
13. Geometric mean	14. Harmonic mean
15. Root mean square level	16. Average integrated rectifier
17. Average power spectrum density	18. Average Welch’s Power
19. Direction of the first slope in the window	20. Number of slope sign changes rectifier in the window

#### Training, validation and testing (non-obstruction)

As shown in Fig. 5, two different machine learning models were used in this study. In model 1, data from every experiment were concatenated and then randomly separated into two different groups of training (60% of data) and testing (40% of data) sets. The training set was further divided into training (85%) and validation (15%) sets. In model 2 (Fig. 5B), data from one randomly chosen experiment was used for testing, while the remaining data was used for training (85%) and validation (15%). The rationale for using the 2 different machine learning models was to (1) build an acceptable classifier and (2) confirm the generality of the classifier by testing with a different set of recording data not previously seen by the training group (i.e., model 2).

Before training the models with data-split as described above, all the training set (not including the 15% validation data) of model 1 was used to find a suitable classifier. K-nearest neighbors (KNN) with coarse kernel^26^, boosted tree with adaptive ensemble method (adaptive boosting, Adaboost)^27^, a neural network (NN) with one hidden layer and 10 units^28^, and support vector machine (SVM) with Gaussian kernel^23^ were all used to perform classification learning. The accuracy of classifiers was compared using the classification accuracy (e.g., confusion matrix) and F_1_-score test. The confusion matrix^42^, also known as an error matrix provided visualization of the performance of an algorithm. Each row of the matrix represented the instances in a predicted class while each column represented the instances in an actual class. The F_1_-score indicated precision and recall of each classifier. The classifier with the highest accuracy as well as F_1_-score was selected as the main classifier for this analysis. Considering that model 1 and model 2 are similar in training and only different in the test set data, classification accuracy and F_1_ score of this section was only reported for model 1 to avoid redundancy.

As a part of the training process, the validation set was used to modify the cost function of the classifier to mitigate any imbalances (e.g., hyper-parameter). The cost function describes the penalty of miss-classification during the optimization/learning process. Due to the asymmetric learned classifier found in this study (i.e., bias towards one of the two classes most likely caused by variation in SNR of recording), the validation set was used to define a weight of miss-classification penalty per class. For both models, a receiver operating characteristic (ROC), the area under the curve (AUC), and a confusion matrix were computed on the test set to analyze the performance.

The ROC curve illustrates the performance of the classification model for the case of a binary classification (i.e. afferent vs efferent) by representing the true positive rate vs. false positive rate for the classifier via the AUC algorithm. The AUC calculates the performance of the classifier across all classification levels (0 ≤ AUC ≤ 1), where 1 denotes a model with 100% correct classification predictions. In general, applying an AUC is useful in showing the effect of each classifier among all the classes. All machine learning processes were done in MATLAB (Mathworks Inc., Natick, MA, USA).

#### Testing and validation the algorithm using obstruction data

Both model 1 and model 2 were used to perform hypothesis testing on the tetrapolar electrode. ENG data obtained during obstructions were provided to both models for prediction and testing of the classifiers which was then compared to the baseline (pre-obstruction) ENG data. This obstruction data was tested for cases of intact nerve, proximal transection, and distal transection (Table 1). It is noted that none of the obstruction ENG data was used in the training process of either model 1 or model 2. Therefore, this data was considered as a secondary method for classifier testing.

### Statistical analysis

All experimental data were summarized as the mean ± SE. A non-parametric test (Wilcoxon rank sum) was applied to determine statistical significance, where p values < 0.05 were considered significant. This analysis was performed using MATLAB (Mathworks Inc., Natick, MA, USA).
